# Medical Certificates for Insurance Purposes

**Published:** 1917-10-20

**Authors:** 


### Medical Certificates for Insurance Purposes.

Are medical superintendents of Poor-Law institutions justified in refusing to issue medical certificates in the precise form desired by Insurance Committees? The point has arisen in Lambeth. Some months ago the Guardians had under consideration the question of the form of medical certificate issued to insured persons in the infirmary to enable them to claim sickness benefit under the National Insurance Act, the Local Government Board having intimated that it considered that the Poor-Law Institutions Order, 1913, required the medical officer to give an inmate of an institution on his request a certificate in which the nature of his illness or other cause of the medical officer's attendance on him was stated. Then the Guardians' clerk expressed the opinion that the Local Government Board was in error in quoting the Poor-Law Institutions Order in this connection and it was decided so to inform the Board, furnishing the reasons upon which the clerk based his opinion, and to inquire whether there was any Order which the Board considered required the medical officer of the infirmary to act as suggested. So far the Board has not answered this query, but the Guardians have had a letter from the National Health Insurance Commissioners with reference to the continued refusal of the medical officer of the infirmary to issue a certificate in the form desired and requesting to be furnished with the observations of the Guardians.

